# TMPRSS4 as an emerging potential poor prognostic factor for solid tumors: A systematic review and meta-analysis

**DOI:** 10.18632/oncotarget.10153

**Published:** 2016-06-17

**Authors:** Ping Zeng, Peng Zhang, Li-Na Zhou, Min Tang, Yi-Xin Shen, Jun Jin, Ya-Qun Zhu, Min-Bin Chen

**Affiliations:** ^1^ Department of Radiotherapy and Oncology, Kunshan First People's Hospital Affiliated to Jiangsu University, Kunshan, Jiangsu Province, China; ^2^ Department of Orthopedics, the Second Affiliated Hospital of Soochow University, Suzhou, China; ^3^ Department of Radiotherapy and Oncology, The Second Affiliated Hospital of Soochow University, Institute of Radiotherapy & Oncology, Soochow University, Suzhou, China

**Keywords:** TMPRSS4, tumor, prognosis, overall survival, time to tumor progression

## Abstract

Recent studies have investigated the potential prognostic value of the transmembrane protease serine 4 (TMPRSS4) in various solid tumors. Yet, the results are inconclusive. Here, we performed this meta-analysis to clarify this issue. Relevant articles were identified by searching PubMed, Web of Science and Embase databases. The primary outcome endpoints were patients' overall survival (OS) and time to tumor progression (TTP). Twelve studies involving 1,955 participants were included. We showed that high TMPRSS4 expression in tumor tissues was significantly associated with patients' poor OS (pooled HR = 2.981, 95% CI = 2.296-3.869, *P* < 0.001) and short TTP (pooled HR = 2.456, 95% CI = 1.744-3.458, *P* < 0.001). A subgroup analysis revealed that the association between TMPRSS4 and the outcome endpoints (OS or TTP) was also significant within China region. We conclude that TMPRSS4 overexpression in solid tumors is associated with patients' poor prognosis. TMPRSS4 could be a valuable prognosis biomarker or a promising therapeutic target of solid tumor.

## INTRODUCTION

The transmembrane protease serine 4 (TMPRSS4) is a single-pass type II membrane protein and a novel serine protease [1]. It contains a serine protease domain, a scavenger receptor cysteine-rich domain and a low-density lipoprotein receptor class A domain [1]. Several TMPRSS4 specific substrates have been identified thus far, including hemagglutinin of the influenza virus (a protein that is vital for virus infection) [2] and the urokinase-type plasminogen activator (uPA), the latter is important for cancer cell invasion [1, 3].

Existing evidences have reported an important role of TMPRSS4 in cell motility, invasion, proliferation and tumor metastasis [1, 4–13]. It has been implied that TMPRSS4 could be an emerging potential therapeutic target in cancer [1]. ITMPRSS4 reduced migration and invasion of lung and colon cancer cells [9, 14]. Knockdown of TMPRSS4 by targeted shRNA inhibited proliferation of lung cancer cells [15]. Reversely, forced overexpression of this protease enhanced lung cancer cell migration and invasion [9]. At the molecular level, TMPRSS4 overexpression was shown to activate several important pro-cancerous signalling cascades, including focal adhesion kinase (FAK), ERK12, Akt, Src and Rac1 pathways [7]. Among them, FAK and Rac1 activation was required for TMPRSS4-mediated cancer cell invasion and epithelial to mesenchymal transition (EMT) [7]. Meanwhile, PI3K or Src inhibitors were shown to inhibit invasiveness of TMPRSS4-overexpressing cells [7].

Growing evidences have demonstrated that TMPRSS4 is overexpressed in multiple solid tumors, including non-small cell lung cancer [6, 9, 15–17], malignant thyroid neoplasm [18–20], breast cancer [21, 22], and colon cancer [5]. Further, multiple studies have implied that elevated TMPRSS4 expression in tumor tissues was correlated with poor survival of cancer patients [1]. The results of those individual studies were however not conclusive. In the present study, we performed this comprehensive meta-analysis to clarify the prognostic value of TMPRSS4 in solid tumors.

## RESULTS

### Demographic characteristics

**Figure 1:**
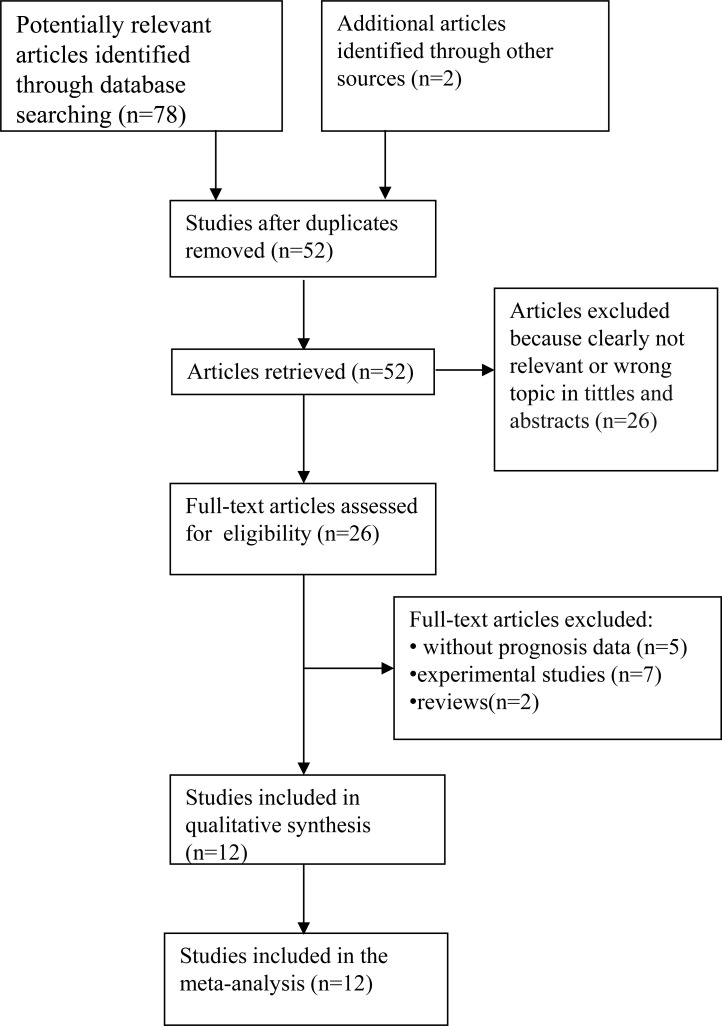
The flow chart of the selection process in the meta-analysis

A total of 80 articles were retrieved by a literature search from PubMed, Embase, and Web of Science databases, using different combinations of key terms. As indicated in the search flow diagram (Figure. 1). Twelve studies reported at least one outcome endpoint and were included in this meta-analysis [16, 21–31]. All studies were assessed by Newcastle-Ottawa Scale (NOS). The quality scores ranged from 6 to 8, indicating that the methodological quality was high. The main features of these eligible studies were summarized in Table 1. In total, the 12 studies provided a sample of 1,955 patients to assess the relationship between TMPRSS4 expression in solid tumor tissues and prognosis of affected patients. The median sample-size was 122, with a range from 69 to 436. Among all cohorts, China (n = 10) became the major source region of literatures, followed by Japan (n = 1) and Spain (n = 1). As for the cancer type, 2 studies evaluated breast cancer, 2 studies evaluated lung cancer, 2 studies study evaluated gastric cancer, 1 study evaluated colorectal cancer, 1 study evaluated prostate cancer, 1 study evaluated salivary adenoid cystic carcinoma, 1 study evaluated cervical squamous carcinoma, 1 study evaluated gallbladder cancer, 1 study evaluated hepatocellular carcinoma. Of all the studies, 11 studies focused on OS, and 8 studies focused on TTP.

**Table 1:** Characteristics of studies included in the meta-analysis

First author	Year	Country	case	Cancer type	Disease Stage	Detection	Provided information on cutoff value	outcome endpoints	NOS score
Chikaishi Y [16]	2016	Japan	161	lung adenocarcinoma	IA-IIIB	ICH	positively stained tumor cells>50%	OS	8
Villalba M [25]	2016	Spain	79	lung squamous carcinoma	I-II	ICH	The H-score was >the median	OS, RFS	7
Wang CH [31]	2015	China	398	hepatocellular carcinoma	I-III	ICH	Score≥4(rang of 0-12)	OS, RFS	8
Wu XY [32]	2014	China	97	gallbladder cancer	I-IV	ICH	Score≥4(rang of 0-9)	OS	7
Sheng H [33]	2014	China	200	gastric cancer	I-IV	ICH	Score≥2(rang of 0-3)	OS	7
Shi G [37]	2014	China	69	prostate cancer		ICH	Score≥9(rang of 0-12)	DFS	6
Cheng D [21]	2013	China	72	Triple-negative breast cancer	I-III	ICH	Score≥3(rang of 0-6)	OS, DFS	7
Cheng D [34]	2013	China	87	cervical squamous carcinoma	I-IV	ICH	Score≥3(rang of 0-7)	OS, DFS	7
Dai W [35]	2013	China	125	salivary adenoid cystic carcinoma	I-IV	ICH	Score≥3(rang of 0-6)	OS, DFS	7
Huang A [36]	2013	China	122	colorectal cancer	I-IV	ICH	Score≥4(rang of 0-9)	OS, DFS	7
Liang B [22]	2013	China	109	breast cancer	I-III	ICH	Score≥3(rang of 0-6)	OS, DFS	8
Luo ZY [38]	2013	China	436	gastric cancer	I-IV	ICH	Score≥2(rang of 0-3)	OS	7

ICH : Immunohistochemistry; NOS: Newcastle-Ottawa Scale; DFS: disease-free survival; RFS: recurrence-free survival.

### Evidence synthesis

**Figure 2:**
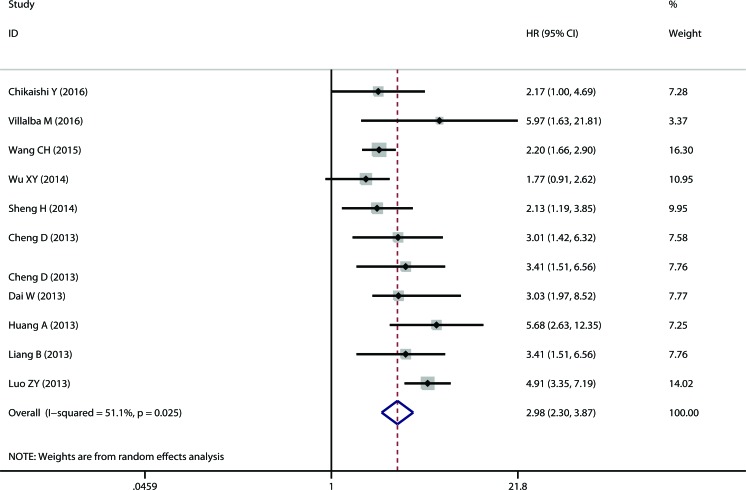
The correlation between TMPRSS4 expression and overall survival (OS) in solid tumor TMPRSS4 overexpression in solid tumors is significantly associated with patients' poor OS (pooled HR = 2.981, 95% CI = 2.296-3.869, *P* < 0.001).

**Figure 3:**
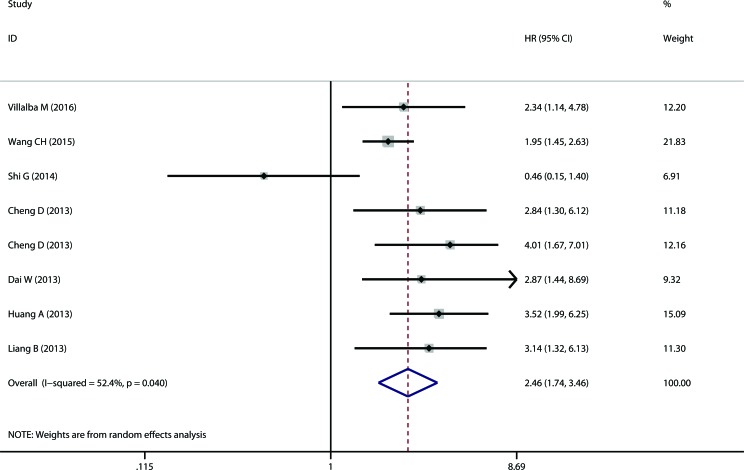
The correlation between TMPRSS4 expression and time to tumor progression (TTP) in solid tumor TMPRSS4 overexpression in solid tumors is significantly associated with patients' short TTP (pooled HR = 2.456, 95% CI = 1.744-3.458, *P* < 0.001).

The meta-analysis of TMPRSS4 expression was therefore based on two outcome endpoints: OS and TTP. Eleven studies were included in the meta-analysis of OS. A random effects model was applied to calculate the pooled hazard ratio (HR) and 95% confidence interval (CI). The heterogeneity test reported a P value of 0.025 and an *I*^2^ values of 51.1%. The results showed that TMPRSS4 overexpression was associated with poor OS of solid tumors (pooled HR = 2.981, 95% CI = 2.296-3.869, P<0.001) (Figure 2). Eight studies included in the meta-analysis reported TTP. A random effects model was again utilized to calculate the pooled HR and 95% CI. The heterogeneity test reported a P value of 0.040 and an *I*^2^ values of 52.4%. The results again demonstrated a significant association between TMPRSS4 overexpression and short TTP (pooled HR = 2.456, 95% CI = 1.744-3.458, P<0.001) (Figure 3). Among all the studies, China is the major source region. We therefore calculated the associations between TMPRSS4 overexpression and outcome endpoints (OS and TTP) within China region. Significant correlations were observed in either OS (pooled HR = 2.985, 95% CI = 2.245-3.969, P<0.001) or TTP (pooled HR = 2.466, 95% CI = 1.663-3.458, P<0.001) in the random-effects model with moderate heterogeneity (*I*^2^ = 57.2%, P=0.016; *I*^2^ = 59.2%, P=0.023, respectively).

### Publication bias and sensitivity analysis

**Figure 4:**
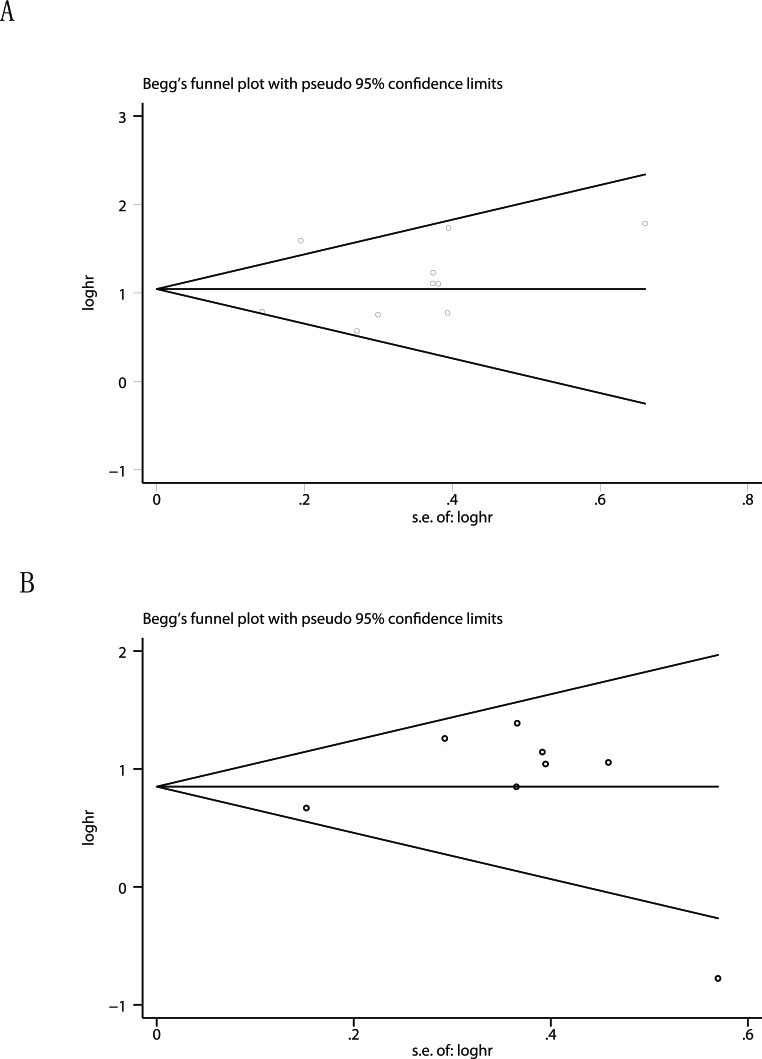
Begg's funnel plots for the studies involved in the meta-analysis of TMPRSS4 expression and the prognosis of patients with solid tumors **A.** Overall survival. **B.** Time to tumor progression. Abbreviations: loghr, logarithm of hazard ratios; s.e., standard error.

**Figure 5:**
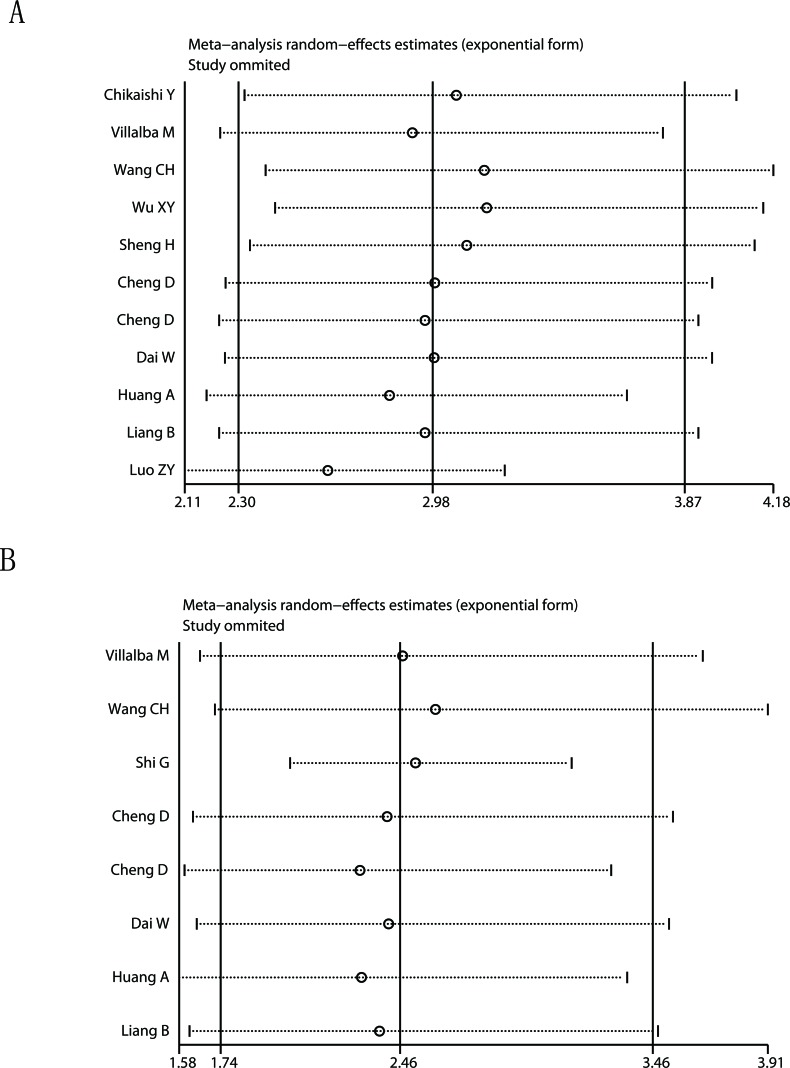
Sensitivity analysis of the meta-analysis **A.** Overall survival. **B.** Time to tumor progression.

Begg's funnel plot and Egger's test were used to estimate the publication bias of the included literatures. The shapes of the funnel plots for the OS and TTP showed no evidence of obvious asymmetry (Figure 4), and Egger's tests revealed non-significant values (P =0.374 and 0.798, respectively). Moreover, sensitivity analysis was carried out to assess the influence of individual study on the overall results of OS and TTP. No individual study dominated this meta-analysis, and the removal of any single study had no significant effect on the overall conclusion (Figure 5).

## DISCUSSION

High TMPRSS4 expression could promote cancer progression and is implied as a poor prognoses marker in solid tumor patients [1, 10, 13, 21, 22, 27]. However, up to now, no meta-analyses have been performed to evaluate the prognostic value of TMPRSS4 expression in the tumor patients. To the best of our knowledge, this meta-analysis is the first comprehensive assessment of the literatures studying TMPRSS4 expression and tumor prognosis. We systematically evaluated survival data for 1,955 solid tumor patients of 12 independent studies. Our results indicated that the expression of TMPRSS4 is a poor prognostic factor of solid tumor, which is negatively associated with patients' OS (pooled HR = 2.226, 95% CI = 1.798-2.655, P<0.001) and TTP (pooled HR = 2.248, 95% CI = 1.745-2.751, P<0.001). Meanwhile, the subgroup analysis revealed that the associations between TMPRSS4 overexpression and poor OS or short TTP were again significant within the China region.

There are several important implications in this meta-analysis. First, high TMPRSS4 expression could be a general poor prognostic marker in solid tumors. We included eight different cancer types, including lung cancer [16, 23], breast cancer [21, 22], gastric cancer [26, 31], hepatocellular cancer [24], prostate cancer [30], salivary adenoid cystic cancer [28], cervical squamous cancer [27], gallbladder cancer [25]. The pooled results from these different types of cancer demonstrated that high TMPRSS4 expression was significantly associated with patients' poor OS and short TTP. This finding can be extended to all solid tumors [16, 21–31]. Second, we demonstrated that high TMPRSS4 expression is correlated with poor OS and short TTP in the Chinese region. Unfortunately, we could not calculate the same associations within other regions due to the limited number of studies. Therefore, additional other region studies analyzing TMPRSS4 expression and cancer prognosis are needed to verify our results. Finally, it emphasizes the potential of TMPRSS4 as a valuable therapeutic target and prognostic biomarker for solid tumor.

There are also some limitations in this meta-analysis that should be considered in interpreting the outcomes. One of the main limitations is the moderate heterogeneity with the studies. However, we used a random-effects model with the pooled data. The heterogeneity among these studies could be explained by the different patients' characteristics or differences in the specific study designs in different tumor types. Furthermore, most of the included studies were designed as retrospective studies, and such studies are more likely to be published if they have positive results than if they have negative results. Therefore, our estimate of the association between increased TMPRSS4 expression and outcome endpoints could have been overestimated. Additionally, the methodology for assessing TMPRSS4 expression and definition of TMPRSS4 positivity are inconsistent.

Taken together, the results clearly demonstrate that high TMPRSS4 expression in solid tumor tissues is associated with poor survival of affected patients. TMPRSS4 could be a useful prognostic biomarker or a promising therapeutic target for solid tumors [1]. However, further studies analyzing specific tumor types and perspectives are needed to further verify the clinical significance of TMPRSS4 expression in solid tumors.

## MATERIALS AND METHODS

### Publication search

PubMed, Embase, and Web of Science databases were searched (up to April 10, 2016) using the search terms: ‘TMPRSS4’, ‘transmembrane protease serine 4’ and “cancer” / “tumor” / “neoplasm” / “carcinoma”. All potentially eligible studies were retrieved and their bibliographies were carefully scanned to identify other eligible studies. Additional studies were identified by searching the references that have cited the original studies. When multiple studies of the same patient population were identified, we included the published study with the largest sample size.

### Inclusion and exclusion criteria

Studies included in this meta-analysis had to meet all of the following criteria: (a) Evaluation of TMPRSS4 expression for predicting prognosis in human cancer; (b) Studies reporting survival data; (c) Studies with adequate data of pooled hazard ratios (HRs) and 95% confidence intervals (CIs), and (d) Studies published in English. Inadequate survival data for further quantification or the follow-up duration shorter than 3 years, letters to the editor and abstracts, as well as case reports, review articles, experimental studies and commentary articles were excluded.

### Data extraction and methodological quality assessment

Information was carefully and independently extracted from all eligible publications by two independent authors. Two outcome endpoints, disease-free survival (DFS) and recurrence-free survival (RFS), were combined, and an unified prognostic parameter, time to tumor progression (TTP), was utilized. The meta-analysis of TMPRSS4 expression was therefore based on two outcome endpoints: overall survival (OS) and TTP.

The following data were extracted for each study: the first author's surname, publication year, country of origin, number of patients analyzed, types of cancer, disease stage, type of detection, score for TMPRSS4 assessment and cut-off values to determine TMPRSS4 positivity. Data for OS and TTP were extracted from tables with respect to TMPRSS4 expression. The Newcastle-Ottawa Scale (NOS) was applied to evaluate the methodological quality, which scored studies by the selection of the study groups, the comparability of the groups, and the ascertainment of the outcome of interest [32]. The studies with 6 scores or more were considered as high quality studies. For studies that presented only Kaplan-Meier curves, Engauge Digitizer version 4.3 was used to extract the survival data. The estimated HRs and 95% CIs were calculated by Tierney's method [33]. Any potential disagreements between the authors were resolved by discussions until a consensus was reached.

### Statistical analysis

Using the data collected from each eligible study, we performed the meta-analysis to evaluate the relationship between solid tumor's TMPRSS4 expression and patients' prognosis. Pooled HRs and 95% CIs for two outcome endpoints (OS, TTP) were calculated via a fixed effects model or random effects model. Heterogeneity assumption was checked using the Q test, and a P values of >0.10 indicated a lack of heterogeneity among studies. We also quantified the effect of heterogeneity using *I*^2^ =100%×(Q - df)/Q. *I*^2^ values of <25% may be considered “low”, values around 50% may be considered “moderate”, and values of >75% may be considered “high” [34]. In the absence of statistical heterogeneity, a fixed effects model was employed to calculate the pooled HRs, otherwise the random effects model was applied [35]. Funnel plots and the Egger's test were employed to estimate the possible publication bias [36]. We performed sensitivity analysis by omitting each study or specific studies to find potential outliers. Statistical analyses were conducted using Stata 14.0 (StataCorp, College Station, TX). P values for all comparisons were two-tailed and statistical significance was defined as P<0.05, unless otherwise mentioned.
